# Zinner Syndrome in a Young Male: A Case Report and Review of the Literature

**DOI:** 10.7759/cureus.74909

**Published:** 2024-12-01

**Authors:** Satyanarayana Kummari, Mahipal Ranga

**Affiliations:** 1 Radiodiagnosis, All India Institute of Medical Sciences, Nagpur, Nagpur, IND; 2 Radiodiagnosis, MNR Medical College and Hospital, Sangareddy, IND

**Keywords:** cystic dilatation of seminal vesicle, ejaculatory pain, male urogenital tract, mesonephric duct abnormality, unilateral renal agenesis, wolffian duct anomaly, zinner syndrome

## Abstract

Zinner syndrome is an extremely uncommon congenital anomaly of the male urogenital tract. It is attributed to an embryological anomaly that arises in the distal segment of the mesonephric or Wolffian duct. It is the inadequate migration of the ureteric bud that contributes to the failure of differentiation of the metanephric blastema, which ultimately results in ipsilateral renal agenesis and atresia of the ejaculatory duct. This condition is characterized by a triad of unilateral renal agenesis, ipsilateral seminal vesicle cysts, and ipsilateral obstruction of the ejaculatory duct. In this case report, we describe a 19-year-old male patient who arrived at the general medicine outpatient department with a dull, aching pain in his abdomen and pelvis, accompanied by a feeling of fullness in his pelvis. He had no history of dysuria, hematuria, or ejaculatory pain. The ultrasound scan, computed tomography, and magnetic resonance imaging of the abdomen and pelvis revealed the absence of the right kidney and compensatory hypertrophy of the left kidney. Another well-defined oblong cystic lesion was seen involving the right seminal vesicle without any enhancement in the post-contrast study. Following a discussion regarding the condition of the patient and radiological findings with the urology team, a diagnosis of Zinner syndrome was established. This was subsequently communicated to the patient, who was presented with various treatment options. He expressed a preference to manage his present relatively mild symptoms and opted for follow-up evaluations. The current case report is intended to highlight a very rare case of Zinner syndrome. In addition, a short overview of the scientific literature was taken into consideration with regard to the specifics of the developmental defect, the primary symptoms, the imaging features, and the potential treatment options.

## Introduction

Zinner syndrome is an extremely uncommon congenital anomaly of the male urogenital tract. The incidence of this condition has been reported to be 0.046% [1]. It is attributed to an embryological anomaly that arises in the distal segment of the mesonephric or Wolffian duct during the four to 13 weeks of gestation. It is the inadequate migration of the ureteric bud that contributes to the failure of differentiation of the metanephric blastema, which ultimately results in ipsilateral renal agenesis and atresia of the ejaculatory duct. This condition is characterized by a triad of unilateral renal agenesis, ipsilateral seminal vesicle cysts, and ipsilateral obstruction of the ejaculatory duct [1,2].

The majority of patients present with local perineal pain, unspecified genitourinary symptoms, or impaired reproductive abilities between the second and fourth decade of life. Pain in the abdominal region, the perineum, and the pelvis are among the most frequently experienced symptoms. Additional potential symptoms include ejaculatory pain, dysuria, hematuria, and urinary tract infections, as well as manifestations of epididymitis and prostatitis [3,4].

## Case presentation

A 19-year-old male patient arrived at the general medicine outpatient department with a dull aching pain in his abdomen and pelvis, accompanied by a feeling of fullness in his pelvis. He had no history of dysuria, hematuria, or ejaculatory pain. The clinical examination revealed pain on palpation of the pelvis. Laboratory investigations like urine analysis, prostate-specific antigen, and a complete blood picture were advised, and they were found to be within normal limits. The patient was then instructed to undergo an ultrasound of the abdomen and pelvis, which revealed a well-defined oblong anechoic lesion involving the right seminal vesicle. The lesion was not separable from the right superior aspect of the prostate. The left seminal vesicle was normal. There was a non-visualization of the right kidney and an enlarged left kidney. The left kidney was normal in shape and echotexture. There was no history of prior surgical intervention.

**Figure 1:**
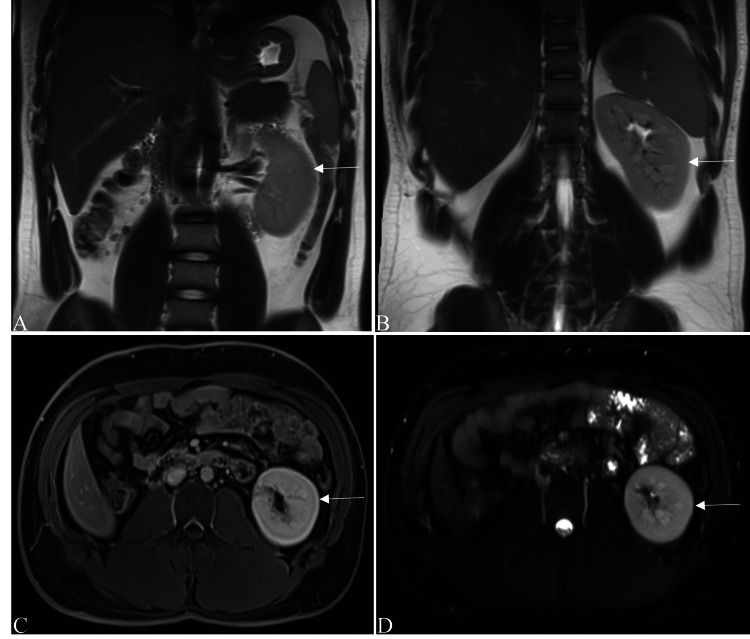
MRI of the abdomen. (A, B) Coronal T2W images, (C) axial post-contrast T1W image, and (D) axial STIR image showed non-visualization of the right kidney and compensatory hypertrophy of the left kidney (white arrows). T1W: T1-weighted; T2W: T2-weighted; STIR: short tau inversion recovery.

**Figure 2:**
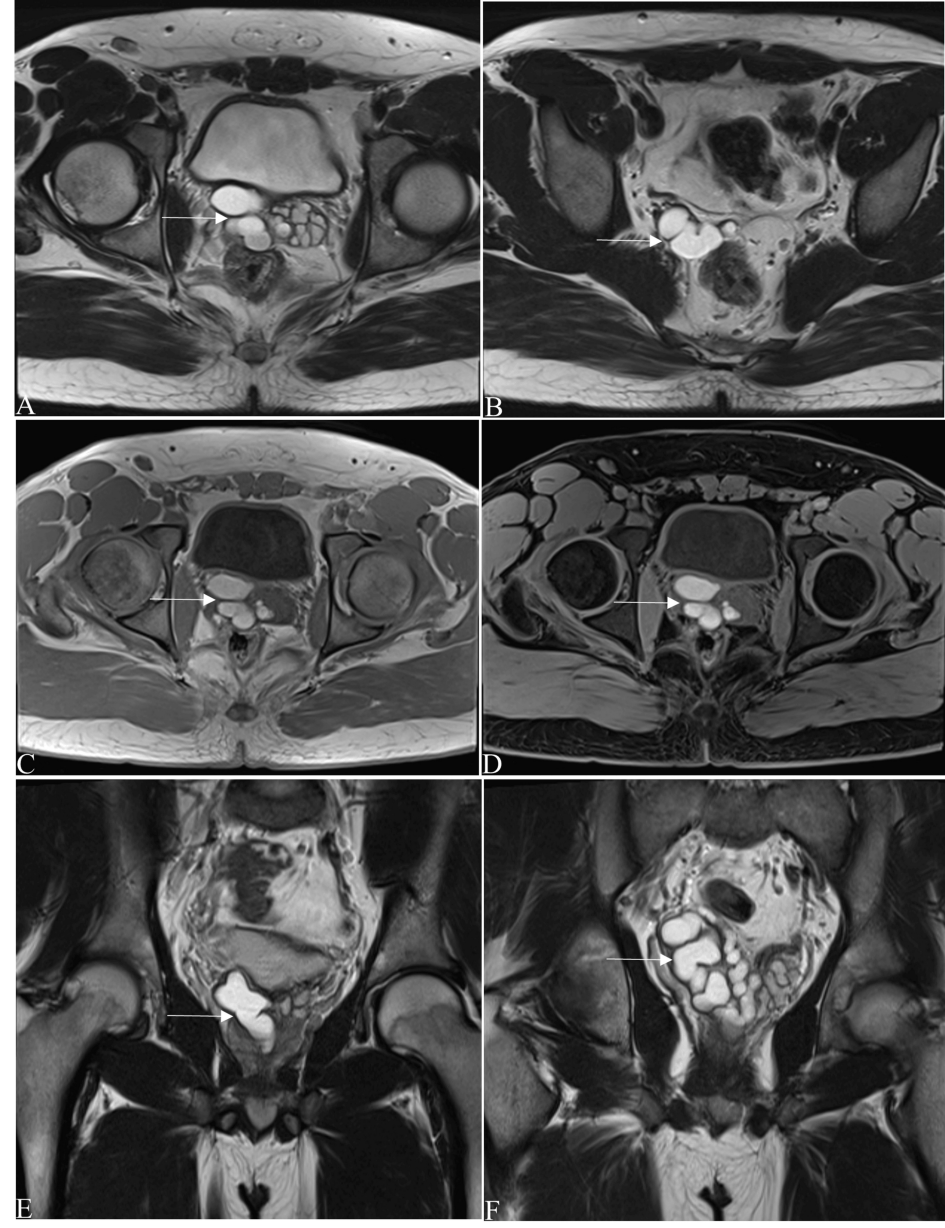
MRI of the pelvis. (A, B) Axial T2W images, (C, D) axial T1W images, and (E, F) coronal T2W images showed a well-defined oblong T1- and T2-hyperintense lesion (white arrows) involving the right seminal vesicle with mass effect on the adjacent structures. T1W: T1-weighted; T2W: T2-weighted.

Cross-sectional imaging was advised for further evaluation. Computed tomography (CT) of the abdomen and pelvis confirmed the absence of the right kidney and compensatory hypertrophy of the left kidney. Another well-defined oblong hypodense lesion (fluid density) was seen involving the right seminal vesicle with a mass effect on the adjacent structures and no enhancement in the post-contrast study. No other abnormalities were found in the rest of the abdominal organs. Magnetic resonance imaging (MRI) of the abdomen and pelvis showed the absence of the right kidney and compensatory hypertrophy of the left kidney (Figure 1) and a well-defined oblong T1-hypointense and T2-hyperintense lesion involving the right seminal vesicle with mass effect on the adjacent structures (Figure 2). The lesion showed no diffusion restriction on the diffusion-weighted imaging sequence and no blooming on the susceptibility-weighted imaging sequence. A post-contrast study showed no enhancement in the lesion. The left seminal vesicle, prostate, and other pelvic structures were normal.

Following a discussion regarding the condition of the patient and radiological findings with the urology team, a diagnosis of Zinner syndrome was established. This was subsequently communicated to the patient, who was presented with various treatment options. He expressed a preference to manage his present relatively mild symptoms and opted for follow-up evaluations every six months, transitioning to annual assessments with ultrasounds while agreeing to consult the urology team should any new symptoms arise. We monitored the patient for more than three years, and he did not report any new symptoms or exacerbations of existing ones.

## Discussion

Zinner syndrome is an extremely uncommon congenital anomaly initially documented by Zinner in 1914. According to the findings of a single pooled investigation that examined 200 patients with Zinner syndrome, the incidence was 0.046% [3]. Zinner syndrome arises from a developmental abnormality of the Wolffian duct that serves as a predecessor to the seminal vesicles and the vas deferens. Additionally, it is responsible for the development of the ureteric bud, which helps to explain the connection between seminal vesicle diseases and renal abnormalities [5-7]. In our particular instance, the patient had an absent right kidney, a right seminal vesicle cyst, and a tortuously dilated right seminal vesicle, which is indicative of right ejaculatory duct obstruction.

Despite the fact that it is frequently asymptomatic, it may manifest itself in the second and third decades of life with symptoms that are not specific to the lower urinary tract or the genital tract [3,4]. The first appearance of symptoms occurs during the years of sexual activity when bodily fluids typically reach the highest level, or when a defective or secondary stenosed duct system prevents appropriate drainage [6,7]. The vast majority of patients experience local perineal pain, unspecified genitourinary symptoms, or impaired reproductive abilities. Additional potential symptoms include ejaculatory pain, dysuria, hematuria, and urinary tract infections, as well as manifestations of epididymitis and prostatitis [3-5].

To properly evaluate these cystic lesions, diagnostic imaging is an extremely important component. One of the first lines of investigation is ultrasound imaging, which can identify cystic lesions as thick or thin-walled cysts that are anechoic in nature with post-acoustic enhancement. The presence of potential internal echoes is something that can be detected in the event of an infection or a hemorrhage into the cyst [6-8]. Both CT and MRI scans are capable of providing a comprehensive examination of the pelvis. However, MRI is the preferred modality for providing a correct anatomical depiction of the male genital tract. Although CT is typically held for additional examination in situations when MRI is either inaccessible or not recommended, the acquisition of contrast material is required to prevent any false interpretations [6-8].

It is necessary to accurately differentiate cysts and the cystic dilatation of seminal vesicles from other pelvic lesions. This encompasses true prostatic cysts, prostatic utricle cysts, ejaculatory duct cysts, Müllerian duct cysts, hydronephrotic pelvic kidneys, bladder diverticula, and ureteroceles. Differentiation is typically predicated on the location (median, para-median, or lateral), intralesional composition, concomitant findings in the urogenital system, and imaging features. Ejaculatory duct cysts and Müllerian duct cysts are located in the middle. The detection of spermatozoa in the aspirate can distinguish seminal vesicle cysts from Müllerian duct cysts. Diverticulosis of the ampulla of the vas deferens and ectopic ureterocele are positioned further laterally [7,8].

Unilateral renal agenesis is characterized by the absence of renal tissue on one side and the absence of an ipsilateral renal artery, with compensatory hypertrophy of the contralateral kidney. A thorough assessment of the remaining abdomen and pelvis must be conducted to confirm the absence of an ectopic kidney, as well as an evaluation of the solitary kidney to ascertain that it does not indicate crossed fused renal ectopia. It is crucial to distinguish between renal agenesis and a small atrophic kidney [5,7]. The diagnosis of renal agenesis depends on imaging investigations to assess the renal anatomy. The limited accessibility of modern imaging technologies in developing nations can hinder the diagnosis of such situations. In this case, ultrasonography, CT, and MRI scans allowed the confirmation of the diagnosis by demonstrating the absence of the right kidney and renal artery.

According to the findings of a pooled review of 200 cases with Zinner syndrome, the most widely accepted treatment options for the condition were observation, aspiration, and surgical operations [3]. The surgical procedures performed included seminal vesiculectomy utilizing various techniques such as open or laparoscopic surgery. The alternative method of treatment is aspiration; however, this method is associated with a significant likelihood of recurrence. Transurethral ejaculatory duct resection is the recommended treatment choice for those who have infertility as a presenting symptom. Asymptomatic small seminal vesicle lesions may be monitored with routine follow-up [3,5,9].

## Conclusions

Zinner syndrome is an extremely uncommon congenital anomaly of the male urogenital tract. It may present with lower abdomen pain in young adult males. It can be diagnosed with a composite of an ultrasound, a CT scan of the abdomen and pelvis, and an MRI scan of the pelvis; however, a thorough review of the images is necessary to rule out potential errors in diagnosis. Patients who are experiencing symptoms can benefit from an MRI for the purpose of planning surgical procedures. In cases when the lesions are relatively small and the condition is asymptomatic, it is recommended to observe the patient and then perform a follow-up ultrasound.
